# I mmunosenescence and Inflammaging: Risk Factors of Severe COVID-19 in Older People

**DOI:** 10.3389/fimmu.2020.579220

**Published:** 2020-10-27

**Authors:** Anna Julia Pietrobon, Franciane Mouradian Emidio Teixeira, Maria Notomi Sato

**Affiliations:** ^1^Laboratory of Dermatology and Immunodeficiencies, LIM-56, Department of Dermatology, Tropical Medicine Institute of São Paulo, University of São Paulo Medical School, São Paulo, Brazil; ^2^Department of Immunology, Institute of Biomedical Sciences, University of São Paulo, São Paulo, Brazil

**Keywords:** COVID-19, coronavirus, aging, immunosenescence, inflammaging, SARS-CoV-2

## Abstract

Old individuals are more susceptible to various infections due to immunological changes that occur during the aging process. These changes named collectively as “immunosenescence” include decreases in both the innate and adaptive immune responses in addition to the exacerbated production of inflammatory cytokines. This scenario of immunological dysfunction and its relationship with disease development in older people has been widely studied, especially in infections that can be fatal, such as influenza and, more recently, COVID-19. In the current scenario of SARS-CoV-2 infection, many mechanisms of disease pathogenesis in old individuals have been proposed. To better understand the dynamics of COVID-19 in this group, aspects related to immunological senescence must be well elucidated. In this article, we discuss the main mechanisms involved in immunosenescence and their possible correlations with the susceptibility of individuals of advanced age to SARS-CoV-2 infection and the more severe conditions of the disease.

## Introduction

Human history is marked by major epidemics, and viral respiratory infections have been major villains in this scenario. The 20th century was certainly marked by the devastating outbreak of the Spanish flu, caused by an influenza A virus of the H1N1 subtype (1). Currently, in the 21st century, coronaviruses appear to show their potential, with three epidemics in the past two decades.

Coronavirus epidemics include severe acute respiratory syndrome (SARS) coronavirus (CoV) (SARS-CoV-1), which occurred between 2002 and 2003, and Middle East respiratory syndrome (MERS)-CoV, which occurred in 2012 (2). Since December 2019, SARS-CoV-2, a new type of coronavirus, has caused respiratory infections ranging from mild to severe clinical conditions and death, and the disease caused by it has been called coronavirus disease 2019 (COVID-19) (3).

The current SARS-CoV-2 outbreak originated in the city of Wuhan in China (4), rapidly spread worldwide and was declared a pandemic by the World Health Organization. By September 20, 2020, COVID-19 had already infected more than 30 million people and caused over 950,000 deaths (5). In this global pandemic scenario, the United States and Brazil are the countries with the highest number of cases and deaths from COVID-19 (5). In Brazil, approximately 51% of SARS cases due to COVID-19 occur in old individuals (over 60 years of age), accounting for 73% of deaths (6).

In fact, it was observed that older people have a higher severity of the disease and are considered the main risk group for COVID-19 (4). This observation has been reinforced by SARS-CoV-2 infection in experimental models, where infected aged Syrian hamsters developed alveolar and perivascular edema (7). A greater severity in individuals of advanced age has also been reported in SARS-CoV-1 (8) and MERS (9). Interestingly, in addition to advanced age, male gender appears to be another risk factor for COVID-19 (10). Additionally, other conditions such as obesity, hypertension, and metabolic diseases are also risk factors for COVID-19 (11).

The following additional four coronaviruses circulate globally among the population: alpha (229E and NL63) and beta (OC43 and HKU1) CoVs. These coronaviruses generally cause mild infections of the upper respiratory tract similar to the common cold (12). However, there are reports of more severe respiratory diseases caused by OC43 and 229E, mainly in older individuals and individuals with chronic immune deficits (13).

Thus far, it is known that cytokine storm in the lungs may be among the immunological components involved in the pathogenesis of COVID-19 in the aged population. Although it has been suggested that alveolar macrophages from older individual have an anti-inflammatory profile, they can develop higher and uncontrolled responses of cellular activation and cytokine production after a pathogen insult and a lower ability to control tissue damage due to infection leaving the lung in a compromised state (14–16). In fact, at baseline state, the lungs of old individuals show increase in levels of complement and surfactant proteins and pro-inflammatory cytokines (15, 16). Interestingly, half of fatal cases of COVID-19 experience a cytokine storm, of which 82% are over the age of 60 (17).

Notably, a series of immunological changes occur with age, causing older individuals to develop immunosenescence (18). These factors can contribute to as pulmonary as systemic exacerbated inflammatory response in older individuals and seem to play a role in increasing susceptibility to respiratory infections.

In fact, a better knowledge of these mechanisms can contribute to the understanding of the infection dynamics in this scenario. Thus, here, we review the main factors related to the senescence of the innate and adaptive immune responses that can be responsible for both the severity and pathogenesis of COVID-19.

## Brief Background of Coronavirus Infection

Coronaviruses have a positive single-stranded RNA genome of approximately 30 kb that forms the viral nucleocapsid with the nucleocapsid (N) protein. This structure is surrounded by an envelope formed of a lipid bilayer in which the spike (S) proteins, membrane (M) protein, and envelope (E) protein are inserted (19).

The coronavirus subfamily consists of four genera, i.e., α, β, γ, and δ coronaviruses, and the α and β genera are responsible for human infections (20, 21). Among the coronaviruses that infect humans, seven are known to cause diseases with flu-like symptoms, but SARS-CoV-1, MERS-CoV, and, more recently, SARS-CoV-2 have gained greater notoriety for their high transmission capacity and severe infections (22–24).

The transmission of coronaviruses occurs mainly through respiratory droplets and close contact between people. Once in the body, the viruses enter target cells when protein S binds to specific input receptors. SARS-CoV-2 S protein binds angiotensin-converting enzyme 2 (ACE2), which is present on the surface of several human cells (25). In addition, several studies have been suggesting that the MERS-CoV receptor, dipeptidyl peptidase 4 (DPP4), can also be used by SARS-CoV-2 during infection (26, 27). The interaction between SARS-CoV-2 and ACE2 recruits the transmembrane protease serine 2 (TMPRSS2), which promotes S protein priming and facilitates viral entry in the host cell (28). Other cellular proteins, such as the protease furin, can also promote SARS-CoV-2 S protein cleavage indicating their potential involvement in viral entrance (29). Once inside the cell, the envelope fuses with the endosomal membrane and releases the viral genome into the cytoplasm where replication and assembly of new viral particles occurs (30).

Coronavirus infection can affect the airways, causing cough, headache, and fever. In more severe cases, the infection can cause tissue damage, especially to the lung tissue, due to the high degree of inflammation generated to fight the virus, leading to the development of pneumonia and dyspnea, which can progress to death (31). Among patients with COVID-19, the highest incidence of severe cases occurs in individuals affected by comorbidities such as lung diseases, diabetes, and hypertension (32–34). Age also appears to be a risk factor for the disease, as worse outcomes and higher mortality rates are observed in older patients (35–37).

## Immunosenescence: Innate Immunity and Susceptibility to COVID-19

### Can Inflammaging Enhance Immunopathogenesis in Old Individuals?

**Figure 1:**
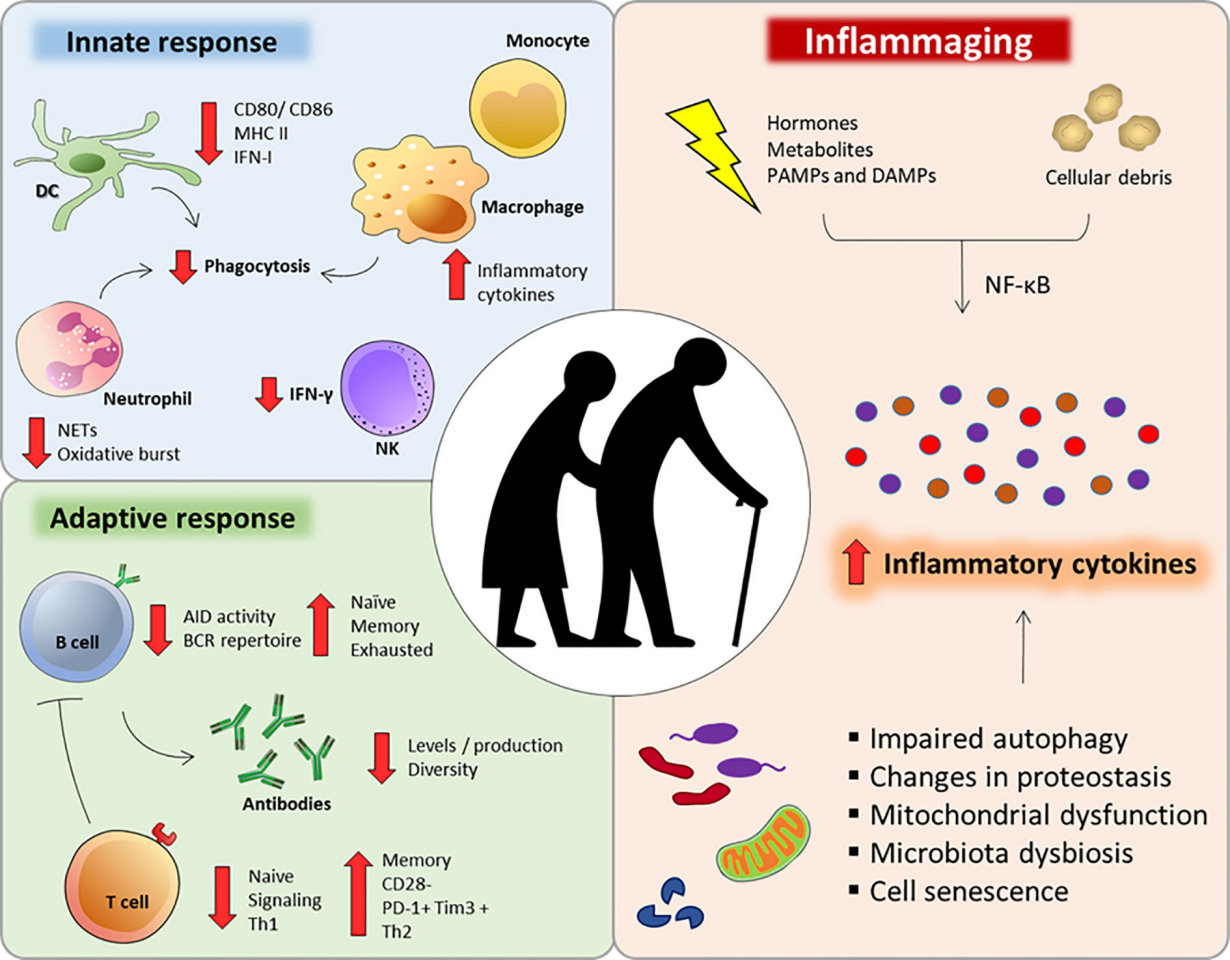
Major immunological alterations observed during immunosenescense. Aging interferes in a number of innate and adaptive immune cells aspects that can impair or compromise their function and response. Additionally, several factors can dysregulate intracellular homeostasis during aging, intensifying the secretion of inflammatory cytokines and chemokines (inflammaging).

The aging process can be understood as a progressive and natural decrease in the biological functions of an organism (18). Despite its enormous plasticity and capacity for renewal, the immune system is also affected during the aging process. Since a functional immune response is essential for maintaining homeostasis and health, the immune aging process, called immunosenescence (**Figure 1**), contributes to the increased susceptibility to infections, cancers and autoimmune diseases (38–40).

A very striking feature of the immunosenescence process is a low-grade proinflammatory state, with an increase in serum inflammatory mediators, such as IL-6, IL-1RA, TNF-α, IL-1, and C-reactive protein (CRP) (41, 42). This low-grade inflammatory state named “inflammaging” is associated with the diminished ability to mount efficient immune responses during the aging process (42) (**Figure 1**).

Inflammaging is caused by a set of hormonal, metabolic and immune factors that constantly provide stimuli that are recognized by innate receptors, favoring an inflammatory environment (43). In addition, senescent cells commonly experience changes in their intracellular homeostasis, including telomeric perturbations and oxidative stress, leading to the activation of signaling pathways such as nuclear factor κB (NF-κB) and increased secretion of cytokines, chemokines, growth factors and lipids (44, 45). This condition in which senescent cells change their secretory phenotype is called the senescence-associated secretory phenotype (SASP) and is a potential contributor to inflammaging (46). The exacerbated inflammatory process associated with age may also be due to a failure to resolve inflammation since many regulatory factors are also deficient in older individuals (47–49).

The inflammatory stimuli that support the phenomenon of inflammaging can be triggered by several factors, including chronic infections and microbiota changes, which are going to be more detailed further in this text. However, sterile components naturally produced during cell cycle can also contribute to this phenomenon. Cellular debris resulting from the cell death process that occurs daily due to chemical and physical stresses as well as the accumulation of metabolic products and cellular catabolic products, such as lipofuscins and beta-amyloid proteins play a crucial role in inflammaging (50, 51). Under the physiological conditions of cell proliferation, such components are usually diluted between dividing cells. However, as the cell proliferation rate reaches its lowest levels due to aging, these molecules accumulate and can be recognized by pattern recognition receptors (PRRs) (52, 53).

In addition, infectious processes during aging can further accentuate the inflammatory condition by releasing pathogen-associated molecular patterns (PAMPs) and damage-associated molecular patterns (DAMPs) (54). During cytomegalovirus (CMV) infection, which infects 40–100% of the population worldwide (55), inflammatory mediators such as prostaglandin E2, IL-6 and TNF-α are released, highlighting the important contribution of this pathogen to inflammaging (56–58). However, a 10-year longitudinal study compared the impact of CMV infection on the serum levels of inflammatory cytokines in 249 individuals and showed that cytokine production in CMV-seropositive and CMV-seronegative individuals is similar (59).

Studies focusing on the current SARS-CoV-2 pandemic have already investigated the association between the pathogenesis of the disease and the inflammatory process. Regardless of the age group, patients affected by COVID-19 have higher plasma concentrations of inflammatory cytokines, such as TNF-α and IL-6, and the chemokines and molecules that activate cells, such as CXCL10, CCL2, CCL3, G-CSF, IL-2, IL-7, and IL-10 (24, 60). There is also a circulating increase in others well-known inflammatory markers, such as CRP, ferritin, D-dimer, and serum amyloid A (SAA) (61–64).

Additionally, *in vitro* SARS-CoV-1 studies have found that the viral cytopathic effect induces apoptosis in Vero E6 and HEK293 cells (65, 66) and that MERS-CoV promotes apoptosis in lung and kidney cells *via* Smad7 and FGF2 (67). A similar effect has also been observed in the HCoV virus (229E) in monocytic cells (68). These findings suggest that target cell apoptosis is a factor contributing to the tissue damage caused during *in vivo* infection. Potential DAMPs released during apoptosis can contribute to the local and systemic inflammatory response by activating PRRs, further aggravating the infection. Additionally, *in silico* studies have indicated that a strong protein-protein interaction exists between the viral S protein and TLR4, a PPR, suggesting that SARS-CoV-2 directly activates proinflammatory pathways (69).

One of the main intracellular pathways resulting from the activation of PRRs is NF-κB, which is the main pathway responsible for inducing the inflammatory response and the appearance of the SASP phenotype (70). DAMPs can also signal *via* the NLRP3 receptor, leading to the activation of the inflammasome pathway and secretion of the inflammatory cytokines IL-1β and IL-18 (71). Interestingly, serum IL-18 levels increase with age, indicating that the pathway strongly contributes to inflammaging (72). Higher levels of IL-18 were also observed in the serum of COVID-19 patients and were associated with disease severity and clinical outcome (73). Moreover, monocytes infected *in vitro* with SARS-CoV-2 presented the formation of NLRP3 puncta, and the same could be observed in mononuclear cells isolated from COVID-19 patients, indicating activation of the inflammasome pathway (73). In fact, NLRP3 inhibitors have already been proposed as potential drugs for the treatment of COVID-19 (74).

In addition, the autophagy pathway seems to be directly related to the development and progression of the inflammaging process. This pathway consists of specialized protein machinery that promotes the recycling of cellular content, generating nutrients and energy for maintaining homeostasis (75). Therefore, autophagy contributes to the elimination of the debris and products of cellular metabolism, preventing its recognition by PRRs and the consequent inflammation (76). However, it has been shown that there is a reduction in the activity of the autophagy pathway during aging (77). Additionally, deficiencies in other pathways that regulate proteostasis during aging, such as reduced proteasome activity, contribute to the accumulation of misfolded protein aggregates that can activate inflammatory pathways (78).

Preliminary studies in DAF2 mutant invertebrates, a longevity study model, indicate that silencing autophagy pathway genes reduces life expectancy in these organisms (79). Additionally, in a clinical trial, Mannick et al. (2018) demonstrated that enhancing the autophagy pathway using mTOR inhibitors reduces the incidence of infections in older individuals and promotes the expression of antiviral genes and a better response to vaccination against the influenza virus, corroborating the importance of the autophagy pathway in the immune response and fighting infections in individuals of advanced age (80).

Another consequence of reduced autophagy during aging is a lower rate of mitophagy, which leads to the accumulation of dysfunctional and damaged mitochondria, changes in the respiratory chain and the generation of reactive oxygen species (ROS) (81, 82). Oxidative phosphorylation products, such as ATP and ROS, induce an inflammatory response by activating the inflammasome pathway (83, 84). In an experimental model, it has been verified that the influenza virus induces the production of mitochondrial ROS, contributing to inflammation, higher viral titers and increased neutrophil infiltration in the airways and lungs (85). It has also been found that oxidative stress generated by H5N1 infection induces the formation of oxidized phospholipids, which activate the TLR4-TRIF pathway in pulmonary macrophages, inducing the inflammatory response (86). In fact, in the context of COVID-19, it was recently shown that mitoquinol and N-acetyl cysteine, two antioxidant drugs, prevented SARS-CoV-2 infection in human primary monocytes (87).

Additionally, mitochondrial lesions generated by stress lead to the release of DAMPs, such as mitochondrial DNA (mtDNA) rich in CpG motifs and bacterial DNA, and, therefore, can activate the inflammatory response *via* TLRs, NLRs and cGAS (88, 89). In this context, a positive correlation was found between the increase in mtDNA and proinflammatory cytokines such as TNF-α, IL-6 and CCL5 during aging (90).

Furthermore, it has been speculated that mitochondrial dysfunctions could be involved in the older population’s greater susceptibility to viral infections since the functioning of MAVS, a protein that assists the RIG-IRF-IFN cascade located in the mitochondrial membrane, depends on the integrity of the mitochondria and oxidative phosphorylation (91, 92). SARS-CoV-1 infection induces mitochondrial fission and MAVS degradation, suppressing the host’s antiviral response (93).

The intestinal microbiota can also play an important role in modulating the proinflammatory response during aging (94, 95). Over time, the composition and diversity of the microbiome changes, leading to dysbiosis in the host and a predominance of Th1-type responses (95, 96). Simultaneously, there is an increase in intestinal permeability with aging, favoring bacterial translocation and inflammaging (95). It has been observed that centenary individuals have a greater prevalence of opportunistic bacteria with proinflammatory characteristics in the intestinal microbiota and a reduced number of bacteria with anti-inflammatory properties. These data are strongly correlated with the serum levels of inflammatory cytokines such as IL-6 and IL-8, suggesting that the microbiota also contributes to the maintenance of inflammaging (97).

Some studies suggest that an exacerbated immune response is mainly responsible for the worsening of SARS-CoV-1 and MERS-CoV infections by contributing more to tissue damage than the actual infection, regardless of the age group (98). Regarding COVID-19, these effects do not seem to be any different. Excessive immune activation and production of proinflammatory cytokines are commonly observed in patients with COVID-19 (99). This exacerbated immune response involving high levels of cytokine release is known as cytokine storm syndrome. Although inflammatory responses are crucial for pathogen clearance, uncontrolled immune responses can be destructive by leading to systemic inflammation, vascular hyperpermeability, multiple organ failure and eventually death (100). In viral infections that reach the lungs, cytokine storm syndrome contributes to apoptosis in epithelial and endothelial cells, leading to fluid leakage in the lungs, the accumulation of leukocytes and tissue fibrosis (101), which, in turn, cause ARDS (102).

**Figure 2:**
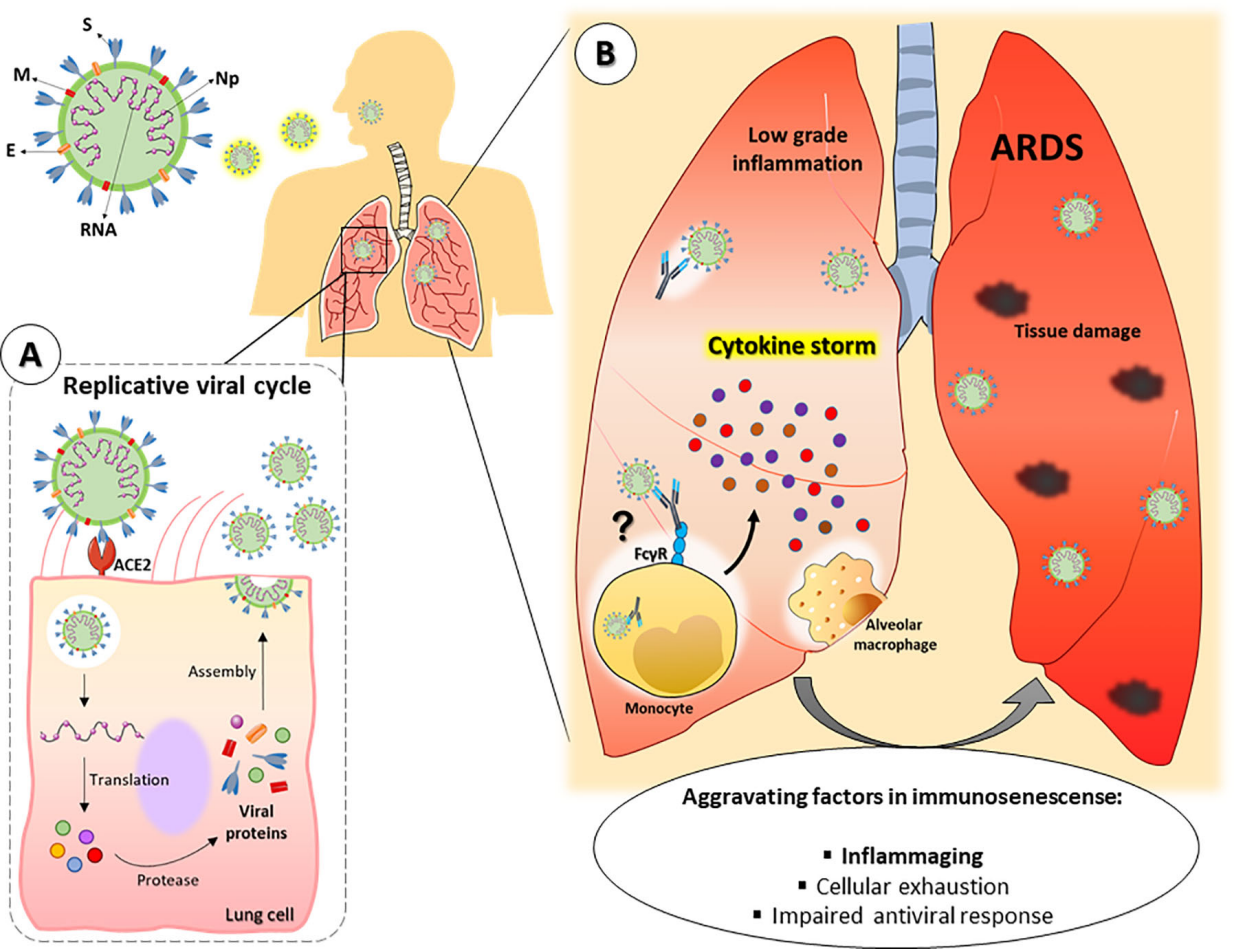
Hypothetical framework of SARS-CoV-2 pulmonary infection in old individuals. SARS-CoV-2 consists of single RNA strand and the following proteins: Spike (S), membrane (M), envelope (E) and nucleocapsid (Np). **(A)** After entering the organism, the virus infects lung cells by binding to the receptor angiotensin-converting enzyme 2 (ACE-2) and establishes its replicative cycle releasing new viral particles. **(B)** Older people have a constitutive low-grade proinflammatory state that, along with other peculiarities of the immunosenescence, can favor the cytokine storm syndrome, leading to a faster progression to ARDS and severe manifestations of COVID-19. In addition, tissue resident or lung-infiltrating immune cells (e.g., neutrophils, monocytes and alveolar macrophages) can contribute to disease severity either by dysfunctional responses associated to immunosenescence or by facilitating viral internalization through ADE. ARDS = Acute respiratory distress syndrome. ADE = Antibody-dependent enhancement.

Considering the abovementioned aspects, it is possible that the inflammaging process favors the greater severity of COVID-19 in the aged population (**Figure 2B**). Although experimental reports are still scarce in the literature, several researchers have proposed that inflammaging could contribute to the more severe outcomes of COVID-19 in older patients (10, 103). In fact, Guaraldi and colleagues demonstrated that treatment with tocilizumab, a monoclonal anti-IL-6 receptor antibody, could attenuate COVID-19 severity in patients older than 60 years (104).

### Other Aspects of Innate Immunity That May Favor SARS-CoV-2

The cells of the innate immune system can be quantitatively and qualitatively affected by the aging process. In the case of monocytes, there is a prevalence of nonclassical and intermediate subtypes associated with a lower phagocytic capacity (43). Monocytes from older individuals also secrete less IFN-α, IFN-γ, IL-1β, CCL20 and CCL8 when stimulated with adjuvants of the innate immune response (105), although some studies have suggested that these cells have a greater capacity to secrete proinflammatory cytokines under baseline conditions or after stimulation in older individuals (106–109). Recently, Zheng and colleagues reported an increase in the monocyte population in aged healthy adults, especially classical CD14 monocytes (110). Monocytes from aged individuals have higher expression of inflammatory genes, such as IL1B, TNF and CXCL8, and increased activation of the NF-κB, Toll-like receptor, inflammasome, and MAPK signaling pathways (110).

In the case of infection by SARS-CoV-2, there is a greater production of IL-6 and GM-CSF in the peripheral blood by CD14^+^ CD16^+^ monocytes (61). Additionally, *in vitro* infection of human monocytes with SARS-CoV-2 leads to the production of several inflammatory cytokines, such as IL-6, IL-1β, TNF-α, and IFN-I (87). Moreover, COVID-19 patients of advanced age have more monocytes than younger patients (110). These cells with an inflammatory profile can migrate into the lungs, contributing to the exacerbated inflammatory response and consequent tissue damage characteristic of the pathogenesis of the disease. In fact, because of their inflammatory properties, monocytes have been suggested to be among the main contributors to the disparate severity of COVID-19 in older patients (111).

The changes occurring in immunosenescence also affect antigen-presenting cells (APCs), such as dendritic cells (DCs) and macrophages (112). In both cell populations, antigen presentation is compromised in the old population, possibly due to the lower expression of CD80, CD86 and MHC-II after exposure to a stimulus (113, 114) and a lower production of superoxide anion by macrophages after treatment with IFN-γ (115). *In vitro* studies investigating DCs derived from peripheral blood monocytes show that infection with MERS-CoV induces the expression of MHC-II and CD86 and promotes the production of IFN-γ, CXCL10, IL-12, and CCL5 (116). However, whether DCs from the older people respond similarly to infection by MERS or other coronaviruses is unclear.

In addition, SARS-CoV-1 is capable of infecting monocyte-derived DCs, rendering these cells producers of inflammatory cytokines, such as TNF-α and IL-6, and chemokines, such as CCL2, CCL3, CCL5 and CXCL10 (117). However, it was not possible to identify the production of antiviral cytokines, such as IFN-α, IFN-β, IFN-γ and IL-12p40, which may indicate a possible viral escape mechanism mediated by blocking these pathways. In fact, Hu et al. showed that the SARS-CoV-1 N protein interacts with TRIM25, preventing the generation of IFN-I *via* RIG-I (118). In COVID-19 patients, an imbalanced production of IFN-I has also been reported, and it seems to correlate with disease severity (119, 120). Moreover, SARS-CoV-2 infection elicits reduced expression of IFN-I and interferon-stimulated genes (121). In addition, pretreatment with IFN-α or IFN-β reduced SARS-CoV-2 titers in *in vitro* infection studies (122, 123). In fact, IFN-I administration has shown promising results in COVID-19 patient clinical trials (124). In a phase 2 study, the triple combination of lopinavir–ritonavir, ribavirin and interferon beta-1b was efficient in reducing symptoms, shortening the duration of infection and hospital stay in patients with mild to moderate COVID-19 (125).

Interestingly, in old individuals, the population of plasmacytoid DCs (pDCs), which is among the main mechanisms of fighting viral infections, is reduced and has less capacity for IFN-α secretion when stimulated with influenza virus due to the deficient expression of TLR7 and TLR9 (126–128). Complementarily, aged human monocytes have imbalanced IFN-I and IFN-III production in response to influenza infection due to defective induction of IFN transcription (129). Taken together, these findings suggest that a reduced IFN-I response in advanced age can contribute to poor clinical outcomes of COVID-19.

Another peculiarity that is possibly associated with the greater susceptibility of old individuals to viral infections is the reduced ability of DCs to perform cross-presentation due to dysfunction in mitochondrial activity and changes in the membrane potential of this organelle (130). These data indicate that the greater susceptibility of the advanced age population to infections may be associated with a lower functional capacity of phagocytes to eliminate pathogens and promote adequate activation of the adaptive immune response.

Aging also contributes to changes in alveolar macrophages (131, 132). During aging, there is a reduction in this cell population in the lungs, which is associated with the downregulation of pathways related to the cell cycle and upregulation of pathways associated with the inflammatory response (14). In fact, alveolar macrophages in animals with an advanced age are in a greater state of cellular activation, secrete more proinflammatory cytokines in response to a *Mycobacterium tuberculosis* stimulus and are refractory to an IFN-γ stimulus (16). Additionally, studies using murine models of influenza infection indicate that alveolar macrophages have a lower ability to control tissue damage due to infection (14). In addition, there is lower expression of the CD204 receptor, suggesting a reduced phagocytosis capacity of cellular debris that could contribute to increased tissue damage (14). In an experimental model of infection by coronavirus hepatitis virus type 1 (MHV-1), the depletion of alveolar macrophages contributes to the reduction in mortality and morbidity caused by the infection (133). In fact, SARS-CoV-2 infection in transgenic mice bearing human ACE2 leads to macrophage infiltration into the alveolar interstitium and alveolar cavities (134), and macrophage activation syndrome is associated with severe respiratory failure in COVID-19 patients (135), suggesting that this cell population plays a crucial role in the pathogenesis of the disease.

Neutrophils in old individuals are also affected by the immunosenescence process. During infections in older people, neutropenia may occur due to the lower proliferative capacity of neutrophil progenitor cells when stimulated by G-CSF (136). However, in SARS-CoV-1 infection, an increase in circulating neutrophils and an association between the infiltrates of this cell type in the lung and the severity of the injury have been observed (137). A similar scenario is observed in SARS-CoV-2 infection, where high neutrophil to lymphocyte ratio in peripheral blood have been reported in severely ill patients (138). Besides, lung infiltration of neutrophils was observed in autopsied COVID-19 patients, revealing capillary extravasation and neutrophilic mucositis (139). These findings indicate that neutrophils not only contribute to systemic inflammation in COVID-19 but also play a crucial role in local tissue damage.

Other characteristics of senescent neutrophils include lower microbicidal activity and a deficiency in the phagocytosis of opsonized bacteria, possibly due to a reduction in CD16 and the oxidative burst mediated by Fc-type receptors (140, 141). Some studies even suggest a deficiency in chemotaxis and release of ROS and neutrophil extracellular traps (NETs) in neutrophils in old individuals (140, 142, 143). However, there is a higher release of NETs in COVID-19 patients, and plasma from infected subjects induces NET release in neutrophils from healthy donors, indicating the participation of these cells in the immunopathogenesis of the disease (144).

The natural killer (NK) cell response is also compromised in old individuals. There is a prevalence of NK CD56dim cells, a cell population with high cytotoxic capacity and production of IFN-γ, and a decrease in NK CD56bright cells, which have a high capacity for cytokine and chemokine production (145, 146). NK cells produced in older people also produce less IFN-γ in the absence of stimulation, which helps to explain the greater susceptibility to viral infections during this stage of life (147). In an animal model of influenza infection, a decrease in NK cells in the lungs, with less capacity for IFN-γ production and degranulation, was observed (148, 149). Similarly, clinical observations of patients with COVID-19 revealed a significant reduction in this cell population during SARS-CoV-2 infection (99). In addition, Zheng et al. reported impaired NK function in severe COVID-19 patients, expressing higher levels of the NKG2A receptor, a cellular exhaustion marker, indicating impaired antiviral immunity (150). However, single-cell analysis of lung bronchoalveolar immune cells revealed a significant increase of NK cells in patients with COVID-19 when compared to healthy controls (151). In addition, in a senescent mice model of SARS-CoV-1 infection, NK cells have been shown to migrate to the lungs (152), indicating a possible contribution of these cells in coronaviruses infection pathogenesis.

Taken together, these findings lead us to propose that innate immune cell dysfunction linked to immunosenescence could be involved in the greater COVID pathogenesis in old individuals either by promoting a less efficient response for fighting the infection and/or favoring an exacerbated inflammatory response.

## Immunosenescence: Adaptive Immunity and Susceptibility to COVID-19

### Can Exhausted T Cells Compromise the Cellular Response Against SARS-CoV-2?

Changes due to aging are also present in the adaptive immune response and are associated with the functional impairment of T and B lymphocytes (153). The sum of these changes renders old people vulnerable to new emerging infectious diseases, as recently observed with SARS-CoV-2. The most prominent factor involves a decrease in the number of naïve cells because of thymic involution (154), an increase in memory/exhausted T cells and a reduction in B cell progenitors in the bone marrow (155). Consequently, these changes reflect the cumulative effect of previous and persistent infections in older individuals (156).

Initial studies involving patients with COVID-19 in China have observed decrease in peripheral lymphocytes was observed (24, 32). This lymphopenia was more prominent in the cases with more severe disease, and 42% of these patients were aged ≥65 years (32). SARS-CoV-1 patients also have been reported to have reduced circulating CD4^+^ and CD8^+^ T cells (157, 158). Indeed, in more severe cases of COVID-19, there is a reduction in CD8^+^ T cells (159), which could prevent an adequate cytotoxic response to fight the virus. Taken together, this profile has been proposed as a biomarker for diagnosis (160).

However, recent data from Arunachalam et al. evidences an increase in effector CD8+ T cells population in infected patients in comparison to health donors in an American and Chinese cohort (161). This could reflect the fact that COVID-19 has distinct effects in different population. In addition, the enhancement of effector T cells has been associated with recovery of SARS-CoV-2 infection (162, 163).

Whether the reduction in the number of T lymphocytes in old individuals could be a condition that predisposes such patients to more severe pathogenesis by COVID-19 remains unknown. However, analysis of immune cell sequencing showed that SARS-CoV-2 enhances T cell polarization from naïve to effector cells and that aging promotes the expression of SARS-CoV-2 susceptibility genes, mainly in T cells (110). In addition to lymphopenia, other age-related comorbidities are predictive of severe/critical cases and a high fatality rate during COVID-19 (37).

Individuals of advanced age have an increase in memory T cells with oligoclonal expansion and a decrease in the T cell receptor (TCR) repertoire (164, 165). These senescent T cells are mainly characterized not only by a low proliferative potential after activation but also by a shortening of telomeres and low telomerase activity, high production of ROS and constitutive activation of p38 MAP kinase, which once activated, blocks signaling *via* TCRs (166). Therefore, the inhibition of p38 MAPK could restore the proliferation and activation of telomerase in senescent T cells.

Phenotypically, senescent T lymphocytes can be identified by the expression of surface markers (CD28^-^, CD27^-^, CD57^+^ and CD45RA^+^) (167–169). In old individuals, the decrease of CD28 has been linked to persistent antigenic stimulation, and with each cycle of proliferation, its expression on the cell surface decreases (170). In addition, telomere shortening occurs, characterizing the process of replicative senescence in T lymphocytes (171).

CD28^+^CD27^+^ undifferentiated T cells have long telomeres, while highly differentiated or senescent CD28^-^CD27^-^ cells have shortened telomeres (172). This phenotype (CD28^-^) is also observed in persistent human immunodeficiency virus (HIV), CMV infections and chronic inflammatory diseases such as rheumatoid arthritis (171). Under these conditions, a persistent antigenic stimulus occurs that leads to replicative senescence.

Several studies indicate that senescent T lymphocytes express the exhaustion molecules PD-1^+^ and Tim3^+^, a phenotype also observed in infections by lymphocytic choriomeningitis virus (LCMV), HIV and HCV (173). Exhausted cells have a low functional capacity, which could prevent the adequate cellular response to the virus, favoring viral escape and intensifying the pathogenesis of COVID-19 in old individuals. We base this hypothesis on studies showing that in SARS-CoV-2 infection, CD4^+^ and CD8^+^ T lymphocytes also have PD-1^+^ and Tim3^+^ expression, prominently in CD8^+^ T cells (159, 174).

In addition, changes in cytokine expression/secretion contribute to the development of immunological senescence. For example, IL-2 is decreased in old individuals, directly impacting the activation and proliferation of T cells (175, 176), which can lead to changes in the intensity and duration of the immune response and contribute to the immunosenescence process. In addition, senescent T cells also secrete high levels of the proinflammatory cytokines IFN-γ and TNF-α (166).

Regarding CD4^+^ helper T cells, the older people have a lower proportion of IFN-γ/IL-4 produced by memory CD4^+^ T cells, with increased Th2 cytokines and decreased Th1 cytokines, which may be a mechanism compensating for the increase in the proinflammatory state characteristic of the aging process (177). Interestingly, patients with severe COVID-19 (mean age of 61 years) also have decreased T-cell IFN-γ production (178). In addition, it has been shown that there is a lower frequency of memory CD4^+^ T cells producing IL-17 (179).

The functional impairment of the CD4^+^ T cell response contributes to the increase in pathology during influenza infection in old individuals (180). The same seems to be true for COVID-19 infections, since patients affected by the most severe form of the disease (mean age of 50 years) develop pathogenic Th1 lymphocytes that coexpress IFN-γ and GM-CSF and are associated with a hyperinflammatory response in the pathogenesis of the disease (61).

CD4^+^ T cells may also contribute to the production of cytokines in the cytokine storm, which is a main mechanism associated with the pathogenesis of COVID-19 in old individuals (181). In patients with severe COVID-19, CD4^+^ T cells express high levels of OX40 (159), a molecule involved in the production of cytokines by T cells (182).

However, an adequate balance between pro- and anti-inflammatory immune responses is essential for preserving health in old individuals. In fact, in severe cases of COVID-19, the evolution to acute respiratory distress syndrome (ARDS) and respiratory failure is a rapid process, which can occur before adaptive response establishment, emphasizing that excessive innate immunity (such as inflammaging) and inadequate regulatory responses may favor the evolution of the infection.

Regulatory T cells (Tregs) are potentially capable of suppressing the immune response and guaranteeing homeostasis (183). The number of naïve circulating Treg cells decreases while the number of memory Treg cells increases with age (184). Although both are suppressive, these different subtypes act at distinct sites in the body, according their expression of chemokine receptors. In addition increase in memory Treg cells is associated with a poor humoral response to influenza vaccination in older individuals (184). In mice, an increase in Treg cells at the expense of helper T cells has also been observed with age (185). Interestingly, patients with more severe COVID-19 present with fewer Treg cells than patients with less severe COVID-19 (186).

Immunosenescence studies are essential for understanding the greater susceptibility of older people to severe respiratory failure induced by viral infections. The presence of exhausted lymphocytes with a low functional capacity compromises the efficient antiviral cellular response, and changes in regulation favor the inflammatory status. These aspects appear to contribute to the severity of COVID-19 due to the cytokine storm.

### Can Previous Antibodies in Old Individuals Aggravate the Pulmonary Condition of COVID-19?

Another important aspect of immunosenescence associated with the adaptive immune response concerns changes in B cells and the consequent failure of the humoral response. Memory B cells have a limited B cell receptor (BCR) repertoire, leading to a decrease in the humoral response to new antigens, with less efficient antibodies and less avidity (187).

A decrease in the ability to produce high-affinity antibodies in old individuals may result from defects in T cell signaling for the adequate activation of B cells, such as inadequate support mediated by T follicular helper cells (T_FH_) (188). Thus, many vaccines are ineffective in old individuals, rendeing them highly vulnerable to newly emerging pathogens, such as SARS and rapidly evolving viruses, such as influenza (189).

In an experimental model of influenza A infection, compared to young mice, aged mice showed a lower frequency of T_FH_ cells and germinal center B cells, with reduced IgG titers but not IgM titers, but the IgM levels do not seem to depend on age (190). Thus, during the aging process, there may be some intrinsic impairment in B cells that compromises their functionality (191).

COVID-19 cohort studies show that seroconversion is observed on approximately the 10th day after symptom onset by increased IgM and IgG antibodies against the viral proteins N and S (60, 192). An age-dependent increase in the viral load (mean age of 66 years) was observed, but there was no correlation between age and the antibody levels. Interestingly, COVID-19 patients with associated comorbidities show lower levels of specific antibodies than COVID-19 patients without associated comorbidities (192).

A subset of B cells called age-associated B cells (ABCs) **identified in mice** has been closely related to the process of immunological senescence and minimally responds BCR and CD40 binding (193–195). ABCs have the potential to inhibit the growth of B cell precursors through the effects of TNF-α, inducing pro-B cell apoptosis (196, 197).

The transcription factor E47 is involved in the regulation of most B cell functions and is negatively regulated in splenic B cells in aged mice, promoting a reduction in the activation of activation-induced cytidine deaminase (AID) and class-switch recombination (198). In older humans, B cells have an age-dependent lower expression of E47 and AID, an associated decline in the number of memory B cells that have undergone class switching (IgG^+^ or IgA^+^) and an increase in naïve cells (IgG^-^/IgA^-^/CD27^-^) (199).

CD27 expression is related to somatically mutated B cell subsets (200, 201) and accordingly, CD27- and CD27+ B cells represent naïve and memory B cells, respectively. In fact, others studies also found higher number of naïve (CD27^-^) than memory (CD27^+^) B cells in individuals of advanced age (202–204).

Old people also have a reduction in the number of circulating B cells (205). In contrast, it has been observed that a double-negative **(DN)** B cell subtype (IgD^-^CD27^-^), which is the counterpart of ABCs in humans, is increased in the peripheral blood of older individuals (206). These **DN** B cells, also called late memory or exhausted cells, are associated with the failure to respond to the influenza vaccine in old individuals. **DN** B cells show SASP, with greater expression of proinflammatory cytokines (TNF, IL-6, and IL-8) and microRNAs associated with inflammation (miR 155/16/93) and are dependent on metabolic signaling *via* MAPK (207). These cells were increased in cases of chronic inflammation, such as HIV infection (208), and in systemic lupus erythematosus (209). For COVID-19, DN B cells are also significantly increased in severe patients (163, 210, 211) but is still unclear if this conditions is dependent on age.

In addition, recent studies have shown that seronegative healthy donors have SARS-CoV-2-specific CD4+ T cells, albeit at lower frequencies, which is indicative of cross-reactivity due to infection between circulating “common cold” coronaviruses (212–214). However, it is unknown whether the older individual could have previous cross-reactive antibodies to the new coronavirus.

In this context, a humoral immune response mechanism widely proposed to be associated with the severity of COVID-19 is related to the possible presence of a phenomenon called antibody-dependent enhancement (ADE) (215–217). ADE occurs when non-neutralizing antibodies generated in a previous viral infection bind Fcγ receptors (FcγR) present in host cells and promote viral internalization. This phenomenon has already been observed in dengue, yellow fever and HIV infection (218). In fact, ADE has also been demonstrated in other coronavirus infections, such as SARS-CoV-1 and MERS (215, 219). In COVID-19, ADE in phagocytes such as alveolar macrophages and lung-infiltrating monocytes could favor SARS-CoV-2 replication in the lung tissue (**Figure 2B**). In addition, the activation of these phagocytes through FcγR could contribute to the cytokine storm in these patients (220). Considering the decrease in the quality of antibody production in older individuals, it is reasonable to think that ADE could be involved in COVID-19 pathogenesis in advanced age patients.

As previously mentioned, different coronaviruses circulate among the population. Therefore, it is plausible that older people have been more exposed to these circulating viruses throughout their lives, thus generating a greater repertoire of antibodies, which could favor a more severe ADE-dependent COVID (**Figure 2B**). This hypothesis is reinforced since children show less susceptibility to SARS-CoV-2 infection (181) considering that their immune system is still developing and that they have had less time to be exposed to antigens. This hypothesis is also reinforced by the fact that some studies show rapid seroconversion to IgG in some patients with SARS-CoV-2 (221).

ADE can also occur when antibody concentrations decrease as a result of waning immunity, as observed by diluted antibodies for SARS-CoV-1 (219). Thus, high levels of antibodies can neutralize the virus, while subneutralizing concentrations could increase infection (222).

It is worth mentioning that highly neutralizing antibodies, such as those proposed to be generated by some SARS-CoV-2 candidate vaccines (223, 224) or those present in convalescent plasma used as treatment for some COVID-19 patients (225, 226), should not trigger ADE.

The IgG-mediated humoral response could also contribute to more severe pulmonary pathology. Compared to patients who recovered within the first 15 days after the onset of symptoms, the patients who died of SARS-CoV-1 had higher levels and faster development of neutralizing anti-S antibodies (227). In addition, in a nonhuman primate model, the previous presence of anti-S IgG antibodies resulted in more severe acute lung injury, with an increase in inflammatory cytokines (CCL2 and IL-8) and recruitment of monocytes/macrophages in the lung (228). These antibodies appear to promote activation *via* FcγR in these cells since their blockade reduced the inflammatory condition. The role of the virus-specific antibody response in lung injury in the pathogenesis of COVID-19 is still unknown.

The presence of immune complexes (ICs) worsens lung injury in viral infections by H1N1 influenza (229) and respiratory syncytial virus (230). Another severe lung disease has also been associated with IC deposition, which promotes not only FcγR-dependent cell activation but also complement system activation and consequent tissue damage (231). It is known that the aging process predisposes individuals to autoimmunity (232); however, whether the accumulation of ICs in old individuals is related to the severity of COVID-19 is unknown.

ICs have a high molecular weight, can be deposited in vessels and tissues, and can activate the complement system, thereby aggravating inflammation (233). In fact, the SARS-CoV-2 N protein has been shown to promote the activation of the complement system lectin pathway and aggravate lung injury in an animal model (234). In addition, these complement pathways were overactivated in the lungs of COVID-19 patients.

To date, no studies have proven that this senescent proinflammatory profile is dependent on B and T cells or other innate cell types and may in fact contribute to a more severe lung pathology in coronavirus-infected patients by increasing the inflammatory response and tissue injury.

## Conclusions and Future Perspectives

Considering the clinical findings obtained thus far concerning SARS-CoV-2 infection and reports of diseases of a similar etiology, it is evident that the immunosenescence process, particularly the increased production of inflammatory cytokines resulting from inflammaging, plays a role in determining the prognosis of COVID-19 in old individuals. From an immunological perspective, the peculiarities of the immune system of older individuals may contribute to both the deficiency of effector mechanisms essential to fighting viral pathogens and the exacerbated inflammatory response, which can accelerate and intensify lung tissue damage. However, despite the strong evidence presented here, tests that accurately demonstrate the association between immunosenescence and the severity of COVID-19 are essential for assisting the search for treatments and the development of vaccines for this most affected age group.

## Author Contributions

AP and FT contributed equally to this work. All authors contributed to the article and approved the submitted version.

## Funding

This work was supported by the Laboratório de Investigação Médica, Unidade 56, Department of Dermatology, School of Medicine, University of São Paulo, Brazil; Fundação de Amparo à Pesquisa do Estado de São Paulo (FAPESP/n° 2018/18230-6 and no. 2017/18199-9).

## Conflict of Interests

The authors declare that the research was conducted in the absence of any commercial or financial relationships that could be construed as a potential conflict of interest.

## Abbreviations

ABC, Age-associated B cell; ACE2, Angiotensin-converting enzyme 2; ADE, Antibody-dependent enhancement; AID, Activation-induced cytidine deaminase; APC, Antigen-presenting cell; ARDS, Acute respiratory distress syndrome; ATP, Adenosine triphosphate; BCR, B cell receptor; CCL, CC chemokine ligand; CD, Cluster of differentiation; cGAS, Cyclic GMP-AMP synthase; CMV, Cytomegalovirus; CoV, Coronavirus; COVID-19, Coronavirus disease 2019; CpG, Cytosine-phosphate-Guanine; CRP, C-reactive protein; CXCL, C-X-C motif chemokine ligand; DAF2, Dauer formation-2; DAMP, Damage-associated molecular pattern; DC, Dendritic cell; **DN, double-negative B cell;** DNA, Deoxyribonucleic acid; DPP4, dipeptidyl peptidase 4; E47, E47 transcription factor; FGF2, Fibroblast growth factor 2; Fc, Fragment crystallizable; FcγR, Fc gamma receptors; G-CSF, Granulocyte colony-stimulating factor; GM-CSF, Granulocyte macrophage colony-stimulating factor; H1N1, Haemagglutinin-1 neuraminidase-1; H5N1, Haemagglutinin-5 neuraminidase-1; HCoV, Human coronavirus; HIV, Human immunodeficiency virus; IC, Immune complex; IFN-I, Interferon type I; Ig, Immunoglobulin; IL, Interleukin; IRF, Interferon regulatory factor; LCMV, Lymphocytic choriomeningitis virus; MAP, Mitogen-activated protein; MAPK, Mitogen-activated protein kinase; MAVS, Mitochondrial antiviral-signaling protein; MERS, Middle East respiratory syndrome; MHC, Major histocompatibility complex; MHV-1, Murine hepatitis virus type 1; mtDNA, Mitochondrial DNA; mTOR, Mammalian target of rapamycin; NET, Neutrophil extracellular trap; NF-κB, Nuclear factor kappa B; NK, Natural killer; NKG2A, CD94/NK group 2 member A; NLR, NOD-like receptor; NLRP3, NLR family pyrin domain containing 3; OX40, Tumor necrosis factor receptor superfamily, member 4 (TNFRSF4); PAMP, Pathogen-associated molecular pattern; PD-1, Programmed cell death 1; pDC, Plasmacytoid DC; PRR, Pattern recognition receptor; RIG, Retinoic acid-inducible gene; RNA, Ribonucleic acid; ROS, Reactive oxygen species; SAA, Serum Amyloid A; SARS, Severe acute respiratory syndrome; SARS-CoV-1, Severe acute respiratory syndrome coronavirus 1; SARS-CoV-2, Severe acute respiratory syndrome coronavirus 2; SASP, Senescence-associated secretory phenotype; Smad7, Mothers against decapentaplegic homolog 7; STING, Stimulator of interferon genes; TCR, T cell receptor; TFH, T follicular helper cell; Th, T helper cell; Tim3, T-cell immunoglobulin and mucin-domain containing-3; TLR, Toll like receptor; **TMPRSS2, Transmembrane Protease Serine 2;** TNF-α, Tumor necrosis factor alpha; Treg, Regulatory T cell; TRIF, TIR-domain-containing adapter-inducing interferon beta; TRIM, Tripartite motif.

## Glossary

ABC: Age-associated B cell
ACE2: Angiotensin-converting enzyme 2
ADE: Antibody-dependent enhancement
AID: Activation-induced cytidine deaminase
APC: Antigen-presenting cell
ARDS: Acute respiratory distress syndrome
ATP: Adenosine triphosphate
BCR: B cell receptor
CCL: CC chemokine ligand
CD: Cluster of differentiation
cGAS: Cyclic GMP-AMP synthase
CMV: Cytomegalovirus
CoV: Coronavirus
COVID-19: Coronavirus disease 2019
CpG: Cytosine-phosphate-Guanine
CRP: C-reactive protein
CXCL: C-X-C motif chemokine ligand
DAF2: Dauer formation-2
DAMP: Damage-associated molecular pattern
DC: Dendritic cell
DN: double-negative B cell
DNA: Deoxyribonucleic acid
DPP4: dipeptidyl peptidase 4
E47: E47 transcription factor
FGF2: Fibroblast growth factor 2
Fc: Fragment crystallizable
FcγR: Fc gamma receptors
G-CSF: Granulocyte colony-stimulating factor
GM-CSF: Granulocyte macrophage colony-stimulating factor
H1N1: Haemagglutinin-1 neuraminidase-1
H5N1: Haemagglutinin-5 neuraminidase-1
HCoV: Human coronavirus
HIV: Human immunodeficiency virus
IC: Immune complex
IFN-I: Interferon type I
Ig: Immunoglobulin
IL: Interleukin
IRF: Interferon regulatory factor
LCMV: Lymphocytic choriomeningitis virus
MAP: Mitogen-activated protein
MAPK: Mitogen-activated protein kinase
MAVS: Mitochondrial antiviral-signaling protein
MERS: Middle East respiratory syndrome
MHC: Major histocompatibility complex
MHV-1: Murine hepatitis virus type 1
mtDNA: Mitochondrial DNA
mTOR: Mammalian target of rapamycin
NET: Neutrophil extracellular trap
NF-κB: Nuclear factor kappa B
NK: Natural killer
NKG2A: CD94/NK group 2 member A
NLR: NOD-like receptor
NLRP3: NLR family pyrin domain containing 3
OX40: Tumor necrosis factor receptor superfamily, member 4 (TNFRSF4)
PAMP: Pathogen-associated molecular pattern
PD-1: Programmed cell death 1
pDC: Plasmacytoid DC
PRR: Pattern recognition receptor
RIG: Retinoic acid-inducible gene
RNA: Ribonucleic acid
ROS: Reactive oxygen species
SAA: Serum Amyloid A
SARS: Severe acute respiratory syndrome
SARS-CoV-1: Severe acute respiratory syndrome coronavirus 1
SARS-CoV-2: Severe acute respiratory syndrome coronavirus 2
SASP: Senescence-associated secretory phenotype
Smad7: Mothers against decapentaplegic homolog 7
STING: Stimulator of interferon genes
TCR: T cell receptor
TFH: T follicular helper cell
Th: T helper cell
Tim3: T-cell immunoglobulin and mucin-domain containing-3
TLR: Toll like receptor
TMPRSS2: Transmembrane Protease Serine 2
TNF-α: Tumor necrosis factor alpha
Treg: Regulatory T cell
TRIF: TIR-domain-containing adapter-inducing interferon beta
TRIM: Tripartite motif
